# Real-World Evidence of Treatment Outcomes in Small Cell Lung Cancer: A Bayesian Mixed Effects and Competitive Risk Approach

**DOI:** 10.2196/84042

**Published:** 2026-04-10

**Authors:** Luca Marzano, Asaf Dan, Adam S Darwich, Luigi De Petris, Salomon Tendler, Rolf Lewensohn, Jayanth Raghothama, Sebastiaan Meijer

**Affiliations:** 1 KTH Royal Institute of Technology, Hälsovägen 11C, Huddinge, Stockholm, 141 57, Sweden, 46 730647775; 2 Department of Oncology and Pathology, Karolisnka Institute, Stockholm, Sweden; 3 Thoracic Oncology Center, Karolinska University Hospital, Stockholm, Sweden; 4 Department of Medicine, Memorial Sloan Kettering Cancer Center, Ner York, NY, United States

**Keywords:** real-world data, small cell lung cancer, SCLC, real-world evidence, adverse effects, survival, toxicity, chemotherapy

## Abstract

**Background:**

Small cell lung cancer (SCLC) is a challenging disease to treat due to rapid progression, development of chemoresistance, and discrepancies in outcomes between real-world data and clinical trials. There is a lack of comprehensive analyses in other studies with regard to intermediate events and the treatment process, such as treatment decisions, progression of disease, and the occurrence of adverse events (AEs) over time.

**Objective:**

The aim of this study was to apply advanced statistical methods to a longitudinal SCLC dataset in order to identify factors of importance for the risk of AEs and for survival.

**Methods:**

Treatment pathways of 421 patients with SCLC collected from Karolinska University Hospital, located in Stockholm, Sweden, between 2016 and 2022, were analyzed with data-driven modeling. The analysis focused on the impact of dose adjustment on AEs, including neutropenia, by estimating odds ratios (ORs) using Bayesian mixed effects modeling. Covariates’ effects on Eastern Cooperative Oncology Group performance status (ECOG PS) deterioration and early discontinuation of chemotherapy with cause-specific hazard ratios (csHR) were explored using competitive risk models. This approach was applied to patient cohorts receiving combinatorial first-line platinum/etoposide and second-line platinum/etoposide or platinum/irinotecan.

**Results:**

At the end of the first-line treatment, most patients exhibited tumor regression (n=167). Patients with neutropenia had longer overall survival (hazard ratio 0.70, 95% CI 0.53-0.92). Higher etoposide dose levels were associated with subsequent occurrences of AEs (OR 5.97, 95% CI 1.41-30.5) and neutropenia (OR 3.55, 95% CI 1.03-13.3). Dose adjustment did not affect overall survival if the patient completed the 4-dose regimen treatment. With regard to second-line therapy, fewer patients completed 4 treatment cycles, and the most common reason for early discontinuation was tumor progression (n=72, 58%). Male patients (n=118) experienced fewer AEs and better first-line treatment response compared to females (csHR 0.51, 95% CI 0.25-0.90). High-risk patients (defined as ECOG PS 2‐3 or age >75 years) with early discontinuation of therapy had survival outcomes similar to those who did not receive any therapy.

**Conclusions:**

Our results indicate that first-line therapies may benefit from more individualized dosing strategies. It would also be beneficial to assess the risk-benefit of treating specific subgroups, including patients receiving second-line therapy. Real-world data proved beneficial for studying therapy response and risk-benefit of treating patient groups that are underrepresented in clinical trials.

## Introduction

Small cell lung cancer (SCLC) is an aggressive and challenging disease to treat, mainly due to the lack of knowledge surrounding chemoresistance mechanisms [1,2]. Standard SCLC treatment consists of defined treatment lines, each comprising 4-6 cycles of platinum-based chemotherapy (cisplatin or carboplatin) with etoposide (PE) or irinotecan (IP). For patients with cancer stages I-III, this is often combined with concurrent thoracic radiotherapy. In stage IV cancer, immunotherapy with checkpoint inhibitors can be combined with chemotherapy, though with a modest median overall survival gain of 2‐3 months in randomized controlled trials (RCTs) and with even smaller survival benefits reported in real-world studies [3,4]. However, most patients with SCLC experience relapse within 6 months after initial treatment, necessitating further therapy, though with limited survival. Real-world studies further indicate that outcomes beyond second-line therapy are poor [5], and the 5-year overall survival for patients with SCLC remains below 10% [6].

In the setting of disease relapse, the distinction between sensitive-relapse and resistance-relapse plays a crucial role in predicting survival. Patients with sensitive disease, defined by relapse occurring more than 90 days after the completion of first-line platinum-based chemotherapy, might be rechallenged with the same chemotherapy regimen administered as first-line and demonstrate significantly better survival outcomes compared to those with resistance disease, where relapse occurs within 90 days [7,8]. In this latter case, the most common approach is to change to other chemotherapy agents if the patient is still in acceptable general condition.

The rapid progression of SCLC often makes it difficult to manage [9], which results in discrepancies in observed outcomes between clinical trials and real-world patients [10]. The knowledge gap between clinical trials and the real-world underrepresented patient populations can be overcome by treatment adjustments based on clinical experience, including choice of chemotherapy agents, dose adjustments, and end-of-life care [9,11,12]. The focus of previous studies has mainly been on survival outcomes, often overlooking patients who received subsequent treatment lines after first-line chemotherapy. Further, there is a lack of studies that analyze events that occur over time in the treatment process [9,13-15].

Real-world data, here defined as health care data produced for the primary purpose of treatment in a clinical setting, offer an opportunity to retrospectively explore the impact of treatment decisions and pathways on patient outcomes, given the variability in the real-world patient population [16,17]. As such, real-world data have the potential to inform the individualization of treatments for specific subpopulations [18,19], such as patients with poor Eastern Cooperative Oncology Group performance status (ECOG PS), older patients, and patients with brain metastases who tend to be underrepresented in clinical trials [10].

However, evidence generation from real-world data is challenging because of complex clinical workflows and issues related to its secondary use, such as inherent biases in data collection and decision-making processes, and nonobservable effects [20,21]. In SCLC treatment, the nuances of dose adjustments, planned treatment pauses, assessment of response, and early discontinuation of treatment due to severe toxicity or health deterioration exemplify the intricacies involved [22,23].

The aim of this study is to describe the time dynamics of patient trajectories in a clinical patient with SCLC cohort in order to identify factors of importance for dose adjustments and survival outcomes. This approach necessitates an appropriate choice of data analysis [24]. While artificial intelligence and machine learning have shown much promise for analyzing real-world data, issues of transparency and interpretability pose a potential risk of propagating biases from the data, including not-captured information and operational confounders [10,25].

Here, the focus was on selecting transparent modeling approaches [25,26], allowing for clinical contextualization and interpretation [10,21]. Then, the analysis consisted of a combination of Bayesian [27-29] and mixed effects models [11,30,31] for studying dose adjustments and their impact on adverse events (AEs; Bayesian logistic regression [32]) and the grade of toxicity (Bayesian cumulative link ordinal regression [32]). Competitive risk Fine-Gray regression [33] was applied to compute cause-specific hazard ratios (csHR) and predict the time course of the therapy, ECOG PS deterioration, and early discontinuation of treatment, defined as discontinuation before completing 4 planned chemotherapy cycles due to AEs, or radiological and clinical signs of tumor progression.

## Methods

### Collection of Longitudinal Data

This study included a total of 421 patients diagnosed with SCLC at the Karolinska University Hospital in Stockholm, Sweden, between 2016 and 2022. Baseline patient characteristics such as age, sex, smoking status, TNM (tumor node metastasis) stage (*International Association for the Study of Lung Cancer, 8th Edition*), Charlson Comorbidity Index, brain metastasis (CNS), and previous cancer prior to SCLC were recorded. ECOG PS was recorded at baseline and at the start and cessation of each treatment line. Blood values, including hemoglobin, creatinine, sodium, lactate dehydrogenase (LDH), C-reactive protein (CRP), and albumin, were computed at baseline and at the start of each treatment line.

The specific drug regimen received by each patient was recorded. Body surface area (BSA) and estimated glomerular filtration rate (GFR) were recorded before the start of treatments. These two variables were used to compute the dose of chemotherapy administered for each patient (eg, GFR for carboplatin dosing using the Calvert formula [34] and BSA for etoposide and irinotecan; see Table S1 in Multimedia Appendix 1). The date and percentage of dose administered for each chemotherapy treatment were registered, including the reason for any modifications or adjustments to the administered doses, for the calculation of dose interval and intensity.

The reason for the dose reduction was recorded. AEs were classified by type and grade of severity of toxicity according to the Common Terminology Criteria for Adverse Events (CTCAE) version 5. In this work, high toxicity was defined as AEs with CTCAE G3-4 [35].

Details on radiotherapy, including the treatment indication (curative, consolidating, as a separate treatment line, or palliative) and fractionation, were reported. At the end of each treatment line, the tumoral response was assessed: complete response, partial response, stable disease, or signs of tumor progression.

The overall survival (OS) was calculated from the date of diagnosis until the patient’s death or the end of the study period, whichever came first. For each treatment line, progression-free survival (PFS) was calculated from the date of the administration of the first dose until signs of progression or death.

### Analysis Description

#### Overview of the Real-World Data Pipeline

Several aspects of the treatment process were analyzed for each treatment line, including dose adjustments due to AEs, changes in ECOG PS from the start to the end of the treatment line, and early treatment discontinuation (stopping treatment before 4 cycles of chemotherapy due to signs of tumor progression, toxicity, deteriorating medical condition, or unwillingness of the patient to receive the treatment) versus no early discontinuation, defined as cessation of a treatment after 4 cycles of chemotherapy due to partial response or stable disease, followed by a pause.

**Figure 1.**
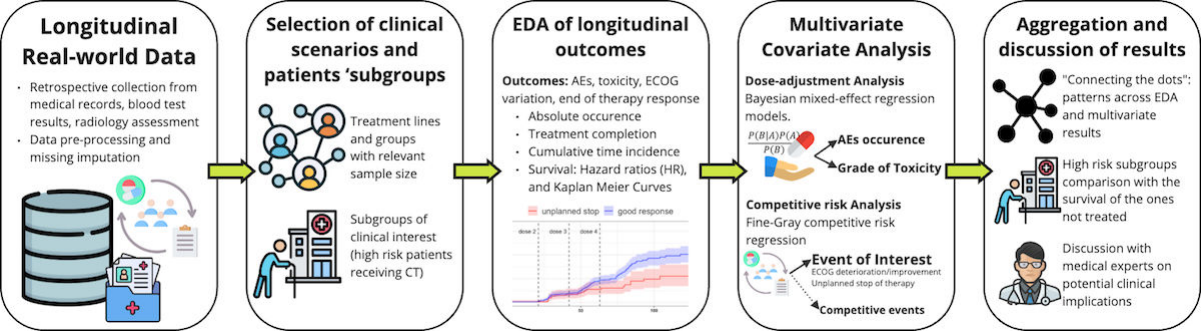
Analytical pipeline description. AE: adverse event; ECOG: Eastern Cooperative Oncology Group; EDA: exploratory data analysis.

The analytical pipeline (Figure 1) can be summarized in the following steps: selection of clinical scenarios and patient subgroups, exploratory data analysis of the longitudinal cohort, multivariate analysis of dose adjustments, multivariate analysis of competitive risks, analysis of high-risk subgroups, and aggregation and discussion of results.

#### Selection of Clinical Scenarios and Patient Subgroups

Prior to commencing the analysis, the collected data were analyzed with a broad perspective to identify key subpopulations of interest. The cohort was explored in terms of baseline characteristics at the first-line treatment decision and the administered chemotherapy lines using summary statistics and Sankey flows for the subsequent treatment lines. Treatment lines with relevant sample size (cohort ≥20 individuals [36]) were detected for the next analysis steps. Moreover, special subgroups of patients receiving treatment but with highly fragile conditions (underrepresented in RCTs) or subjected to a high risk of poor survival outcomes were selected. The continuous covariates with high skewness were log-transformed before starting the analysis. Furthermore, in case of missing values, for each treatment line analysis, values were imputed using the machine learning algorithm MissForest [37]. MissForest was chosen because it is particularly effective for datasets with mixed-type variables and complex interactions, maintaining low imputation errors and preserving relationships among variables even with high percentages of missing information [38].

#### Exploratory Analysis of the Longitudinal Cohort

CTCAE G3-4, ECOG PS change at the end of treatment, and early discontinuation of therapy were assessed by exploring absolute count and relative percentages. Hazard ratios (HRs) and Kaplan-Meier curves were computed to estimate the impact on survival outcomes [39]. The cumulative incidence-time curves of dose reduction due to AEs, separating patients with good therapy response and the ones with unplanned stops, were analyzed [40]. Moreover, the survival outcomes were estimated by stratifying patients by those receiving 4 doses (full course) without dose reduction, patients who received 4 cycles but with dose reduction, and patients who received fewer than 4 cycles.

#### Multivariate Analysis of Dose Adjustments

For the dose adjustment analysis, the association between dose reductions caused by an AE and the grade of toxicity was explored. This was done by training 2 Bayesian mixed-effect regression models: a binary logistic regression for the AE incidence [32,41] and a cumulative link regression for the grade of toxicity modeled as an ordinal outcome [32,42]. The priors used in these Bayesian models were the empirical ones computed from the analyzed cohort. The longitudinal variables (dose level, dose interval, and their mutual interaction) were analyzed with respect to the key baseline characteristics. The Bayesian logistic regression can be summarized as follows:


$$Y_{AE}=log\left(\frac{P\left({AE}_{i+1}\right)}{1-P\left({AE}_{i+1}\right)}\right)=b_{0}+b_{D}D_{i}+b_{\Delta t}\Delta t_{i}+b_{D\Delta t}D_{i}\Delta t_{i}+\sum_{baselines}b_{x}x+\sum_{random}\epsilon$$

where *P*(AE*_i_*_+1_) is the probability of a dose reduction due to an adverse event at dose *i*+1 caused by the *i*th dose *D_i_*, with interval Δ*t_i_* between the doses, *x* baseline covariates with *b* its estimated regression coefficient, and ϵ random effects (unexplained individual variability term). The cumulative link ordinal Bayesian regression can be summarized as follows:


$$Y=log\left(\frac{P\left({GT}_{i+1}\leq j\right)}{1-P\left({GT}_{i+1}\leq j\right)}\right)=b_{0j}+b_{D}D_{i}+b_{\Delta t}\Delta t_{i}+b_{D\Delta t}D_{i}\Delta t_{i}+\sum_{baselines}b_{x}x+\sum_{random}\epsilon$$

With similar notation for the Bayesian logistic regression, where *P*(GT*_i_*_+1_≤*j*) is the probability of grade of toxicity lower than or equal to *j* (with *j*=0,..., 4), and *b*_0_*_j_* is the baseline intercept for the *j*th level. The Bayesian models were trained by running 4 independent Markov chain Monte Carlo simulations with 2500 iterations for each chain. The convergence of the chains was assessed through trace plots, density plots, Gelman-Rubin statistics, and estimates of the Markov chain effective sample size. In case of lack of convergence, the model was retrained by increasing the iterations (see Multimedia Appendix 2). The odds ratios (exponentials of the coefficients *b*) with the 95% CIs were estimated for all the covariates. The random effects were estimated for the individual patients, administered doses, and the dose interval. This allowed for individual differences and the time-dependent nature of the dose variables to be considered.

#### Multivariate Analysis of Competitive Risks

For the variation of ECOG PS at the end of the treatment line and the early discontinuation of the therapy, a competitive risk time-to-event model was developed. Fine-Gray competitive risk regression was used with the outcomes [32], the PFS associated with the investigated treatment line, and the event of interest to estimate the distributed HRs of the covariates. The model can be summarized as follows:


$$h_{j}\left(t\right)=h_{0}\left(t\right)exp\left(b_{1}x_{1}+\ldots +b_{m}x_{m}\right)$$

where *h_j_* is the cause-specific hazard of the event of interest, *h*_0_ is the baseline hazard, and the exponential of the *m*th coefficient *b* is the distributed hazard ratio of the *m*th covariate *x*. The csHRs were computed for ECOG PS deterioration/improvement (in competition with unchanged PS or improvement and deterioration) and the early discontinuation of the therapy in competition with the planned stop of treatment due to partial response or stable disease. Here, we indicate the OS or PFS Cox hazard ratios with HR, csHR for the cause-specific hazard ratios computed with the Fine-Gray model, and OR for the odds ratios obtained by the Bayesian mixed effects models.

#### High-Risk Subgroups Analysis

Multivariate analysis was performed for the subgroups of patients with detected fragile conditions receiving treatment. The OS of these patients was compared to the OS of patients with similar characteristics that did not receive chemotherapy. This allowed the comparison of survival outcomes of patients with poor responses with the ones who did not receive chemotherapy, thus comparing the benefits and risks of the received treatment.

#### Aggregation and Discussion of Results

The considerations obtained during the exploratory data analysis are discussed together with the results obtained from the multivariate analysis. This allows for creating a connection between covariate effects on the longitudinal outcomes that can impact the OS or treatment decision. Moreover, the high-risk subgroups of patients are compared with the ones that did not receive treatment stratified by the significant covariates or longitudinal outcome discovered during the multivariate subgroup analysis to assess the risk-benefit of receiving treatment. Finally, the overall outcomes are discussed with the clinical experts regarding the potential clinical implications.

### Ethical Considerations

The study received ethical approval from the Swedish Ethical Review Authority (approval number: 2023-07389-01). Patients still alive at the time of data collection signed a written informed consent. The data was anonymized prior to use, and the approval covers secondary analysis without additional consent. Research was performed in accordance with the Declaration of Helsinki.

## Results

### Selection of Clinical Scenarios and Patient Subgroups

The cohorts taken into consideration were the patients receiving PE as first-line therapy and the patients receiving either rechallenge with PE or IP as second-line therapy. The analysis included stage III-IV patients and excluded patients with ECOG PS of 4 (n=29). Figure S1 in Multimedia Appendix 3 reports the inclusion/exclusion flowchart of the cohort. In terms of toxicity, only neutropenia was analyzed in detail in this study because it was the most commonly reported treatment-related AE (see Table S2 in Multimedia Appendix 1). Figures S1-S4 in Multimedia Appendix 3 show Sankey flows of the subsequent treatment lines and the dose adjustments.

The identified high-risk subgroups included the first-line patients receiving PE with ECOG PS 2‐3 (n=114), with CNS metastases (n=42), over the age of 75 years (n=99), and with stage IVB (n=135). These subgroups were selected since they are known to be either underrepresented in RCTs or are associated with relatively poor OS compared to the general population [10]. The age threshold was chosen to define a sharp subgroup of older patients from the cohort. The multivariate analysis was not performed for patients with CNS metastases due to wide confidence intervals of ORs and csHRs caused by the small sample size of the subgroup (n=42).

Laboratory covariates referred to throughout the results include hemoglobin, sodium, LDH, CRP, albumin, creatinine, BSA, and GFR. For some individuals, blood values and lab tests were missing (neutrophil counts 44.9%, LDH 22.9%, and CRP 18.3%). The percentages of missing values for neutrophil counts at the start of the treatment lines were as follows: 50.9% in the first-line PE, 34.8% in the second-line PE, and 39.3% in the second line of IP. The high percentage of missing neutrophil values was due to these not being routinely measured at baseline before the initiation of chemotherapy treatment. Therefore, these values were imputed using the MissForest algorithm as described in the Methods section.

A summary of the analyzed cohorts for the first- and the second-line is reported in Table 1.

**Table 1.** Summary of the analyzed cohort.

Covariates	First-line	Second-line	
	Platinum etoposide (n=289)	Platinum etoposide (n=69)	Platinum irinotecan (n=56)
Baselines			
Age (years), n (%)			
<75	190 (65.7)	51 (73.9)	47 (83.9)
≥75	99 (34.3)	18 (26.1)	9 (16.1)
Sex, n (%)			
Female	171 (59.2)	34 (49.3)	29 (51.8)
Male	118 (40.8)	35 (50.7)	27 (48.2)
ECOG PS^a^ baseline, n (%)			
0	58 (20.1)	16 (23.2)	15 (26.8)
1	117 (40.5)	35 (50.7)	29 (51.8)
2	75 (26.0)	13 (18.8)	9 (16.1)
3	39 (13.5)	5 (7.2)	3 (5.4)
Charles Comorbidity Index			
Mean (SD)	8.11 (2.30)	7.22 (2.18)	8.07 (1.87)
Median (min-max)	9 (2-15)	8 (3-12)	8 (3-12)
Missing, n (%)	4 (1.4)	2 (2.9)	2 (3.6)
Brain metastasis, n (%)			
No	247 (85.5)	66 (95.7)	46 (82.1)
Yes	42 (14.5)	3 (4.3)	10 (17.9)
Previous cancer, n (%)			
No	158 (54.7)	50 (72.5)	44 (78.6)
Yes	60 (20.8)	19 (27.5)	12 (21.4)
Missing	71 (24.6)	— 0 (0)	0 (0)
TNM^b^ stage			
Overall stage, n (%)			
IIIA-B	61 (21.1)	19 (27.5)	8 (14.3)
IIIC	19 (6.6)	6 (8.7)	1 (1.8)
IVA	74 (25.6)	20 (29.0)	15 (26.8)
IVB	135 (46.7)	24 (34.8)	32 (57.1)
TNM-T, n (%)			
T1-2	70 (24.2)	16 (23.2)	9 (16.1)
T3	34 (11.8)	9 (13.0)	8 (14.3)
T4	185 (64.0)	44 (63.8)	39 (69.6)
TNM-N, n (%)			
N0	20 (6.9)	9 (13.0)	6 (10.7)
N1	12 (4.2)	3 (4.3)	2 (3.6)
N2	101 (34.9)	24 (34.8)	17 (30.4)
N3	155 (53.6)	33 (47.8)	31 (55.4)
Missing	1 (0.3)	0 (0)	0 (0)
TNM-M, n (%)			
M0	80 (27.7)	25 (36.2)	9 (16.1)
M1a	37 (12.8)	13 (18.8)	6 (10.7)
M1b	37 (12.8)	7 (10.1)	9 (16.1)
M1c	135 (46.7)	24 (34.8)	32 (57.1)
Blood values and weight at the start of the treatment after MissingForrest Imputation			
Hemoglobin (g/L)			
Mean (SD)	130 (16.5)	127 (19.5)	122 (14.1)
Median (min-max)	130 (87-170)	128 (14-156)	120 (100-156)
C-reactive protein^c^ (mg/L)			
Mean (SD)	34.7 (49.6)	10.3 (11.8)	21.8 (38.9)
Median (min-max)	15 (1-331)	7 (1-69)	10 (1-267)
Sodium (mmol/L)			
Mean (SD)	137 (4.97)	138 (4.93)	137 (4.41)
Median (min-max)	137 (112-148)	139 (127-151)	137 (120-146)
LDH^d^^e^ (μkat/L)			
Mean (SD)	26.1 (44.4)	23.4 (46.9)	22.7 (31.5)
Median (min-max)	9.30 (2-607)	10.8 (2.60‐377)	11.2 (3-193)
Creatinine^c^ (μmol/L)			
Mean (SD)	68.6 (22.9)	71.7 (21.9)	68.9 (20.3)
Median (min-max)	66 (26-204)	69 (38-139)	65 (32-123)
Albumin^c^ (g/L)			
Mean (SD)	32.7 (14.3)	34.8 (4.33)	34.1 (7.62)
Median (min-max)	32 (3-250)	35 (24-43)	33 (25-82)
Neutrophil count^c^ (10^9^ /L)			
Mean (SD)	34.8 (44.5)	5.92 (2.66)	6.84 (3.66)
Median (min-max)	19.2 (1.8‐514)	5.59 (0.5‐14.5)	6.23 (0.9‐26.4)
Weight (kg)			
Mean (SD)	70.6 (15.3)	69.8 (12.5)	75.8 (16.7)
Median (min-max)	68.5 (37-125)	70 (45-103)	78 (48-113)
Missing, n (%)	3 (1.0)	2 (2.9)	3 (5.4)
Dose calculation			
Body surface area (m^2^)			
Mean (SD)	1.79 (0.21)	1.84 (0.38)	1.84 (0.25)
Median (min-max)	1.78 (1.18‐2.37)	1.81 (1.31‐4.53)	1.86 (1.09‐2.31)
Missing, n (%)	4 (1.4)	2 (2.9)	1 (1.8)
GFR^e^ (mL/min/1.7)			
Mean (SD)	78.6 (28.7)	78.2 (25.4)	86.4 (41.0)
Median (min-max)	75 (8-235)	75 (30-157)	80 (36-299)
Missing, n (%)	11 (3.8)	1 (1.4)	2 (3.6)
Etoposide dose administration, n (%)			
10 days	69 (23.9)	9 (13.0)	0 (0)
3 days	220 (76.1)	60 (87.0)	0 (0)
Adverse effects of dose reduction			
Neutropenia, n (%)			
No	205 (70.9)	43 (62.3)	53 (94.6)
Yes	84 (29.1)	26 (37.7)	3 (5.4)
Other adverse events, n (%)			
No	225 (77.9)	52 (75.4)	33 (58.9)
Yes	64 (22.1)	17 (24.6)	23 (41.1)
CTCAE^f^ G3-4, n (%)			
No	185 (64.0)	39 (56.5)	38 (67.9)
Yes	104 (36.0)	30 (43.5)	18 (32.1)
End of treatment reported response			
ECOG PS variation, n (%)			
Stable	87 (30.1)	36 (52.2)	28 (50.0)
Deterioration	141 (48.8)	21 (30.4)	17 (30.4)
Improvement	61 (21.1)	12 (17.4)	11 (19.6)
Treatment line response, n (%)			
Early discontinuation	110 (38.1)	36 (52.2)	37 (66.1)
Complete response, partial response, or stable disease	179 (61.9)	33 (47.8)	19 (33.9)
Radiotherapy			
Treatment intention, n (%)			
No radiotherapy	172 (59.5)	47 (56.0)	50 (70.4)
Curative	45 (15.6)	0 (0)	0 (0)
Consolidating	39 (13.5)	4 (4.8)	0 (0)
Palliative	33 (11.4)	30 (35.7)	19 (26.8)
Prophylactic cranial irradiation (PCI)	0 (0)	3 (3.6)	2 (2.8)
Survival outcomes			
Progression-free survival (days)			
Mean (SD)	185 (174)	157 (168)	127 (93)
Median (min-max)	163 (0‐1980)	125 (0‐1170)	110 (9-473)
Missing, n (%)	43 (14.9)	8 (11.6)	4 (7.1)
Overall survival (days)			
Mean (SD)	311 (303)	607 (391)	355 (157)
Median (min-max)	245 (11‐2310)	516 (140‐2310)	318 (144-911)
Status at the last reported information, n (%)			
Dead	262 (90.7)	63 (91.3)	54 (96.4)
Alive (censored)	27 (9.3)	6 (8.7)	2 (3.6)

a ECOG PS: Eastern Cooperative Oncology Group performance status.

b TNM: tumor node metastasis.

c Log transformation was applied during the analysis to handle high skewness.

d LDH: lactate dehydrogenase.

e GFR: estimated glomerular filtration rate.

f CTCAE: Common Terminology Criteria for Adverse Events.

### Exploratory Survival Analysis

In the first-line setting, chemotherapy doses were reduced in 127 out of 289 (43.9%) patients, mainly due to any grade of neutropenia (n=89) and AE G3-4 (n=104) (Table 1). Similar figures were observed for the second-line setting (PE n=69, IP n=56), with neutropenia mainly associated with PE treatment (n=26). At the end of the first-line setting, 179 (out of which 167 partial responses) patients did not experience early discontinuation of treatment. Early discontinuation of therapy happened in 110 cases due to clinical (n=48) or radiological (n=23) signs of disease progression. In the second-line setting, early discontinuation (n=72) was more common than completion of treatment (n=52).

**Figure 2.**
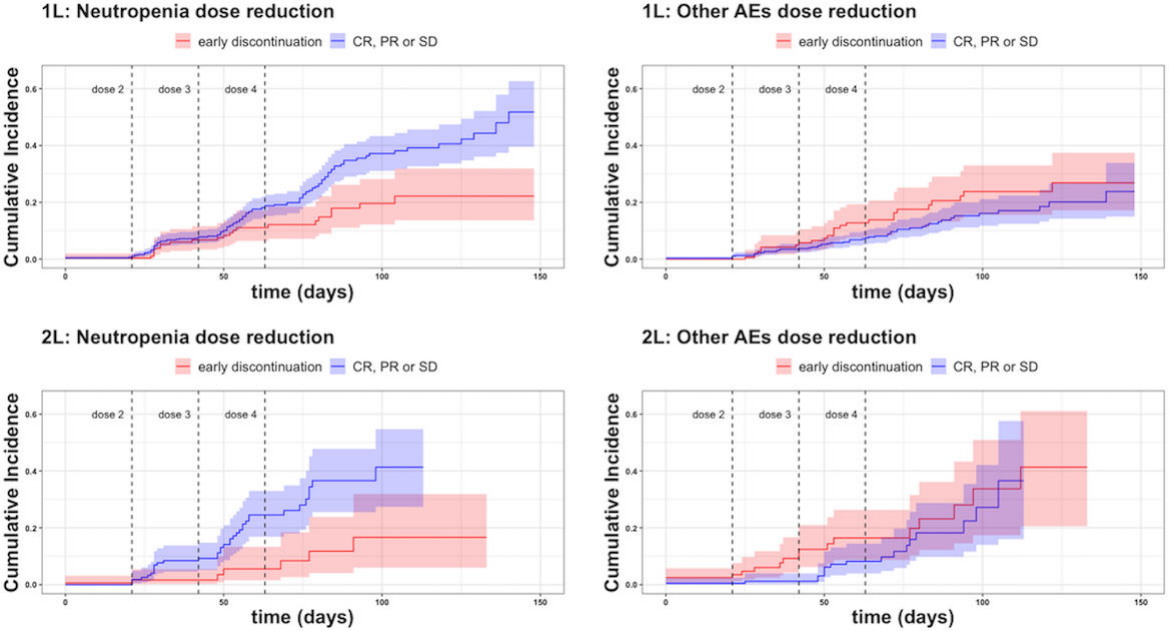
Cumulative incidences of dose reduction (%) due to neutropenia and other adverse events (AEs) for the first- and the second-line therapy. The curves are stratified by early discontinuation of chemotherapy (red curves) and therapy response (CR: complete; PR: partial; SD: stable disease) (blue curves). Dotted lines correspond to the 3-week intervals corresponding to planned administration of the second, third, and fourth doses. 1L: first-line therapy; 2L: second-line therapy.

**Figure 3.**
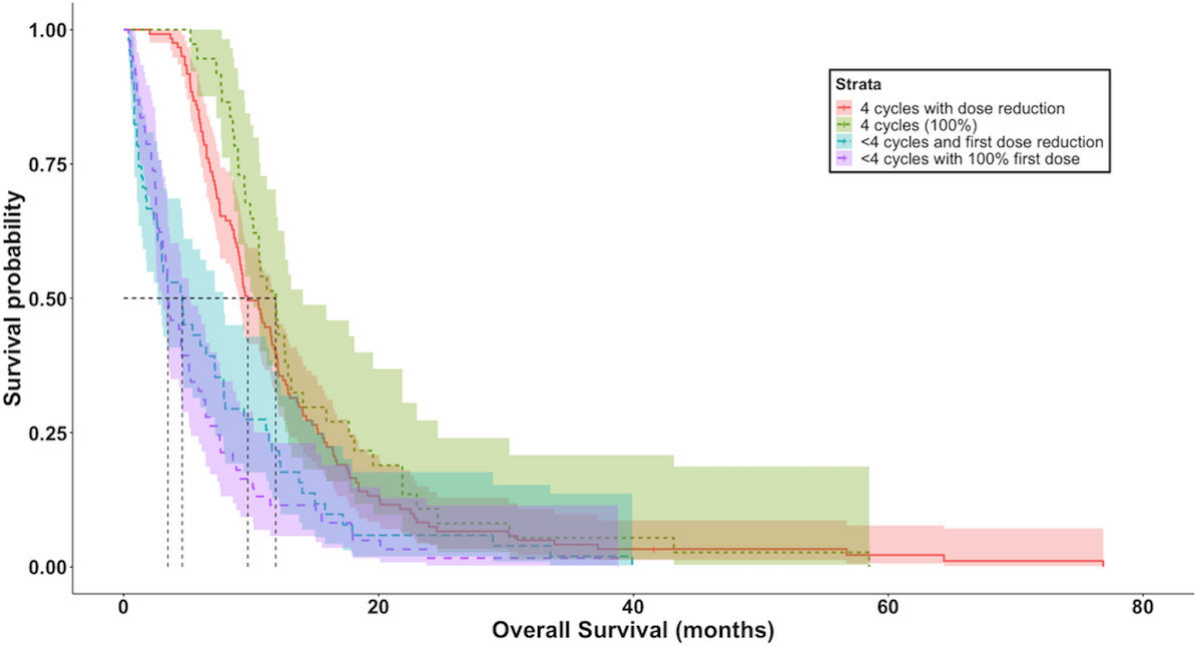
Kaplan-Meier curves for overall survival of patients receiving first-line treatment stratified by administered doses. Patients receiving fewer than 4 doses experienced early discontinuation of the therapy.

Figure 2 shows the cumulative incidences of dose adjustment due to neutropenia. The incidence was higher for patients in both lines who achieved treatment completion without premature discontinuation. In the first-line scenario, occurrences of neutropenia and other AEs were associated with more favorable OS (HR 0.70, 95% CI 0.53-0.92 and HR 0.72, 95% CI 0.57-0.95, respectively). In the second-line scenario, only neutropenia was associated with better OS (HR 0.51, 95% CI 0.33-0.80). OS of patients completing 4 cycles of first-line treatment was not affected by dose reduction due to AEs (HR 0.85, 95% CI 0.59-1.23; reference: patients completing 4 cycles without dose reduction) (Figure 3).

Patients with early discontinuation of therapy in the first-line setting showed an expected markedly worse OS compared to patients who completed 4 cycles of chemotherapy (HR 4.27, 95% CI 3.27-5.57). Similarly, worse OS was observed for patients with early discontinuation during second-line therapy (HR 2.21, 95% CI 1.50-3.27). Moreover, ECOG PS deterioration was significantly associated with a poorer prognosis, even if this was only seen in the first-line setting (HR 2.05, 95% CI 1.54-2.72; reference: stable ECOG PS). These patients resulted in early discontinuation of therapy, of which 45 resulted in PFS less than 3 months (median 61, IQR 43-68 days).

Patients receiving PE as a second line completed the 4-dose cycle more often than the IP patients (73.36% vs 41.07%, respectively). Interestingly, the PFS of PE patients was similar compared to patients receiving IP (HR 0.84, 95% CI 0.57-1.23). However, as expected, second-line PE patients showed better OS (HR 0.51, 95% CI 0.33-0.78) compared to the ones receiving IP, when overall survival was computed from the start of the second-line treatment.

### Multivariate Analysis

#### First-Line Therapy PE

Tables2  3 detail the results of the dose adjustment and the competitive risk analysis for the first-line therapy. All the longitudinal variables presented significant variability (see Table S3 in Multimedia Appendix 1). Interindividual variability was higher than interoccasion variability. This high variability combined with the presence of patients with little longitudinal information (ie, patients receiving only one or two doses before interrupting the therapy) impacted some of the estimates resulting in large intervals.

**Table 2.** Bayesian mixed effects analysis for the first-line platinum etoposide patients.

Covariates	Adverse effects, OR^a^ (95% CI)	Neutropenia grade, OR (95% CI)
Dose interval	1.00 (0.94‐1.05)	0.99 (0.95‐1.03)
Dose platinum	0.60 (0.07‐4.33)	0.56 (0.14‐2.18)
Dose etoposide	5.97 (1.41‐30.5)	3.55 (1.03‐13.3)
Dose platinum × dose interval	1.00 (0.97‐1.03)	1.01 (0.98‐1.05)
Dose etoposide × dose interval	1.02 (0.99‐1.06)	1.04 (1.00‐1.08)
Etoposide dose load		
3 days	—	—
10 days	1.69 (0.31‐9.23)	5.03 (1.25‐23.9)
Weight	0.28 (0.03‐1.88)	1.52 (0.30‐7.47)
Estimated glomerular filtration rate	3.14 (0.47‐24.8)	1.59 (0.47‐5.39)
Body surface area	0.53 (0.04‐5.95)	0.16 (0.02‐1.12)
Hemoglobin	0.77 (0.37‐1.50)	0.88 (0.52‐1.46)
Creatinine	3.98 (1.45‐12.5)	1.32 (0.67‐2.56)
C-reactive protein	0.83 (0.40‐1.76)	0.99 (0.57‐1.72)
Sodium	0.97 (0.50‐1.91)	1.11 (0.68‐1.81)
Lactate dehydrogenase	1.59 (0.78‐3.41)	1.56 (0.91‐2.82)
Albumin	0.61 (0.30‐1.20)	0.83 (0.48‐1.40)
Neutrophil counts	0.39 (0.16‐0.82)	0.67 (0.36‐1.21)
Age (years)		
≥75	—	—
<75	0.92 (0.20‐4.42)	0.77 (0.26‐2.32)
Gender		
Female	—	—
Male	0.27 (0.05‐1.39)	1.96 (0.47‐8.81)
ECOG PS^b^ baseline		
0	—	—
1	0.34 (0.07‐1.56)	0.89 (0.31‐2.61)
2	0.49 (0.07‐3.22)	1.14 (0.29‐4.71)
3	0.45 (0.04‐4.05)	4.24 (0.66‐36.5)
TNM-T^c^		
T4	—	—
T1-2	1.87 (0.44‐8.35)	0.68 (0.23‐1.97)
T3	0.50 (0.07‐3.54)	0.73 (0.19‐3.04)
TNM-N		
N3	—	—
N0	0.84 (0.07‐10.1)	1.92 (0.30‐12.2)
N1	5.65 (0.17‐190)	37.4 (2.46‐721)
N2	0.97 (0.25‐3.86)	1.03 (0.37‐2.61)
TNM-M		
M1c	—	—
M0	6.01 (0.72‐61.9)	1.47 (0.31‐6.97)
M1a	2.95 (0.45‐21.2)	1.14 (0.30‐4.64)
M1b	3.22 (0.43‐25.6)	0.47 (0.10‐2.28)
Smoking status		
Smoker	—	—
Previous smoker	0.92 (0.24‐3.49)	1.11 (0.39‐3.21)
Nonsmoker	0.62 (0.02‐18.5)	1.05 (0.07‐14.8)
Charles Comorbidity Index	1.49 (0.55‐3.92)	1.26 (0.62‐2.63)
Brain metastasis		
No	—	—
Yes	0.70 (0.10‐4.85)	0.86 (0.21‐3.63)
Previous cancer		
No	—	—
Yes	0.66 (0.13‐2.84)	0.58 (0.20‐1.57)

a OR: odds ratio.

b ECOG PS: Eastern Cooperative Oncology Group performance status.

c TNM: tumor node metastasis.

**Table 3.** Competitive risk analysis results for the first-line treatment of platinum etoposide.

	Early discontinuation of treatment				
Covariates	n	csHR^a^ (95% CI)	n	ECOG PS^b^ deterioration, csHR (95% CI)	ECOG PS improvement, csHR (95% CI)
Age (years)					
<75	149	—	140	—	—
≥75	82	1.01 (0.55‐1.84)	64	1.57 (0.75‐3.27)	0.43 (0.14‐1.33)
Gender					
Female	131	—	111	—	—
Male	100	0.51 (0.29‐0.90)	93	0.89 (0.41‐1.96)	0.52 (0.13‐2.04)
ECOG PS baseline					
0	49	—	—	—	—
1	94	1.08 (0.53‐2.19)	—	—	—
2	61	2.93 (1.31‐6.52)	—	—	—
3	27	4.15 (1.68‐10.2)	—	—	—
Charles Comorbidity Index	231	1.10 (0.92‐1.32)	204	0.98 (0.80‐1.22)	1.09 (0.83‐1.45)
Previous cancer					
No	181	—	159	—	—
Yes	50	0.75 (0.38‐1.50)	45	0.77 (0.34‐1.71)	1.34 (0.40‐4.46)
Brain metastasis					
No	196	—	176	—	—
Yes	35	0.83 (0.40‐1.75)	28	0.64 (0.24‐1.71)	1.50 (0.34‐6.50)
TNM-T^c^					
T4	149	—	130	—	—
T1-2	52	1.55 (0.82‐2.93)	47	1.33 (0.67‐2.62)	0.79 (0.29‐2.11)
T3	30	0.78 (0.42‐1.45)	27	0.96 (0.50‐1.85)	0.54 (0.13‐2.20)
TNM-N					
N3	129	—	113	—	—
N0	17	0.17 (0.04‐0.70)	15	0.73 (0.27‐1.98)	0.48 (0.13‐1.81)
N1	8	1.05 (0.36‐3.08)	6	0.13 (0.04‐0.49)	0.00 (0.00‐0.00)
N2	77	0.80 (0.47‐1.36)	70	0.77 (0.40‐1.51)	0.59 (0.22‐1.54)
TNM-M					
M1c	119	—	102	—	—
M0	52	1.52 (0.53‐4.41)	47	0.49 (0.11‐2.14)	1.07 (0.17‐6.67)
M1a	29	1.25 (0.58‐2.69)	25	0.55 (0.18‐1.69)	3.05 (1.04‐8.93)
M1b	31	1.03 (0.53‐1.97)	30	1.21 (0.54‐2.72)	4.26 (0.81‐22.5)
Hemoglobin	231	1.01 (0.99‐1.03)	204	1.00 (0.98‐1.01)	0.99 (0.97‐1.02)
Lactate dehydrogenase	231	1.44 (1.14‐1.82)	204	1.19 (0.94‐1.49)	0.98 (0.58‐1.64)
C-reactive protein	231	1.13 (0.92‐1.38)	204	1.02 (0.80‐1.31)	1.22 (0.89‐1.68)
Neutrophil counts	231	0.99 (0.73‐1.35)	204	1.40 (1.06‐1.85)	0.51 (0.32‐0.82)
Sodium	231	1.02 (0.97‐1.07)	204	1.01 (0.95‐1.07)	1.05 (0.96‐1.15)
Creatinine	231	2.40 (0.84‐6.83)	204	2.54 (0.68‐9.48)	0.80 (0.09‐7.39)
Weight	231	0.99 (0.95‐1.03)	204	1.06 (1.00‐1.12)	0.93 (0.85‐1.02)
Estimated glomerular filtration rate	231	1.00 (0.99‐1.02)	204	1.00 (0.99‐1.01)	0.99 (0.97‐1.01)
Body surface area	231	2.97 (0.14‐62.5)	204	0.01 (0.00‐1.58)	144 (0.07‐286,657)
Etoposide dose load					
10 days	57	—	51	—	—
3 days	174	0.39 (0.22‐0.69)	153	0.72 (0.35‐1.48)	3.65 (0.91‐14.7)
Radiotherapy					
No radiotherapy or palliative	175	—	151	—	—
Curative	24	0.41 (0.14‐1.24)	22	1.35 (0.42‐4.28)	0.31 (0.06‐1.47)
Consolidating	32	0.20 (0.06‐0.69)	31	0.50 (0.16‐1.50)	0.53 (0.24‐1.17)

a csHR: cause-specific hazard ratio.

b ECOG PS: Eastern Cooperative Oncology Group performance status.

c TNM: tumor node metastasis.

The results show a strong association between higher etoposide dose levels and the subsequent occurrence of AEs (OR 5.97, 95% CI 1.41-30.5), specifically neutropenia (OR 3.55, 95% CI 1.03-13.3). Early discontinuation of therapy was associated with higher LDH levels (csHR 1.44, 95% CI 1.14-1.82), baseline ECOG PS 2 (csHR 2.93, 95% CI 1.31-6.52), and baseline ECOG PS 3 (csHR 4.15, 95% CI 1.68-10.20).

Males showed less probability of early discontinuation of the first treatment line compared to females (csHR 0.51, 95% CI 0.29-0.90). As did subgroups of patients with M0 and those receiving consolidating radiotherapy (n=29) (Table 3). Similarly, patients with M1a status regardless of T and N stage were associated with ECOG PS improvement (Table 3).

#### Second-Line Therapy PE-IP

Results of the multivariate analysis for the second-line treatment are reported in Tables S4 and S5 in Multimedia Appendix 1. Due to the large confidence intervals of the ORs, the ORs are reported on a log scale.

A higher dose of platinum was associated with a higher incidence of AEs and a higher risk of neutropenia. Patients receiving IP experienced fewer AEs. Male patients were less likely to experience AEs and neutropenia. However, male patients were associated with worse PFS (HR 1.62, 95% CI 1.10-2.38) due to early discontinuation of the second line, regardless of PE or IP (n=44). Higher sodium levels were instead associated with lower risk of neutropenia and ECOG PS deterioration. Patients with TNM-M M1a had a higher probability of ECOG PS deterioration and more AEs. CNS metastasis was linked to increased risk for ECOG PS deterioration and early discontinuation of therapy.

### High-Risk Subpopulation Analysis: To Treat or Not to Treat?

As defined at the beginning of the Results section, high-risk subgroups included patients with ECOG PS 2‐3, aged ≥75 years, and TNM stage IVB disease. Multivariate analyses were performed separately within each subgroup to identify predictors of toxicity, ECOG PS variability, early discontinuation, and survival outcomes.

The results of these analyses are reported in Tables S6-S11 in Multimedia Appendix 1. Table 4 summarizes the significant covariates for each subgroup. Similar to the second-line therapy analysis, the ORs were reported in log scale due to the wide confidence intervals.

**Table 4.** Qualitative summary of statistically significant covariates identified in the multivariate analysis for high-risk subgroups^a^.

High-risk subgroup	Adverse events occurrence	Neutropenia	ECOG PS^b^ deterioration	ECOG PS improvement	Early discontinuation
TNM-IVB^c^	Albumin (–) Neutrophil counts (–)	Dose × interval etoposide (+) BSA^d^ (–)	Neutrophil counts (+)	Neutrophil counts (–) LDH^e^ (+)	LDH (+) Creatinine (+)
ECOG PS 2‐3	Etoposide 10 days regimen (+) Neutrophil counts (–) Age ≥75 years (+) Male (–)	Dose × interval platinum (+)	Neutrophil counts (+)	Neutrophil counts (–) LDH (+)	Etoposide 10-days regime (+) TNM-N0 (–) TNM-M0 (+) HB^f^ (+) LDH (+) CRP^g^ (+) CCI^h^ (+)
Age ≥75 years	Male (–) ECOG 3 (–) CNS^i^ (–)	Dose × interval etoposide (+) Weight (–) Male (–) TNM-N0 (+) TNM-M1a (+)	TNM-N2 (–)	——	ECOG 2 (+) TNM-N0 (–) LDH (+)

a For each outcome, covariates with statistically significant associations are shown. “+” indicates odds ratio (OR) or cause-specific hazard ratio (csHR) with a direct association with the studied outcome. “–” indicates OR or csHR with an inverse association. Detailed results are reported in Tables S6-S11 in Multimedia Appendix 1.

b ECOG PS: Eastern Cooperative Oncology Group performance status.

c TNM: tumor node metastasis.

d BSA: body surface area.

e LDH: lactate dehydrogenase.

f HB: hemoglobin.

g CRP: C-reactive protein.

h CCI: Charles Comorbidity Index.

i CNS: brain metastasis.

Results observed in the subgroup analyses that aligned with the overall cohort were the consistent association of elevated LDH and ECOG PS 2‐3 with an increased risk of early discontinuation of therapy across all subgroups.

In patients with TNM stage IVB and in patients aged ≥75 years, a higher etoposide dose was associated with an increased risk of neutropenia, while older male patients (aged ≥75 years) and male patients with ECOG PS 2‐3 were associated with a lower risk for AEs. For patients with ECOG PS 2‐3, the platinum dose was associated with an increased risk of neutropenia, while the etoposide 10-day regimen was associated with an increased risk of AEs.

The comparison with patients who did not receive chemotherapy showed that, although the occurrence of AEs was associated with longer survival in the overall cohort, patients with ECOG PS 2‐3 or aged ≥75 years who experienced early discontinuation had HRs closer to those of the nontreated subgroup rather than to those patients who achieved disease control (Table S12 in Multimedia Appendix 1).

**Figure 4.**
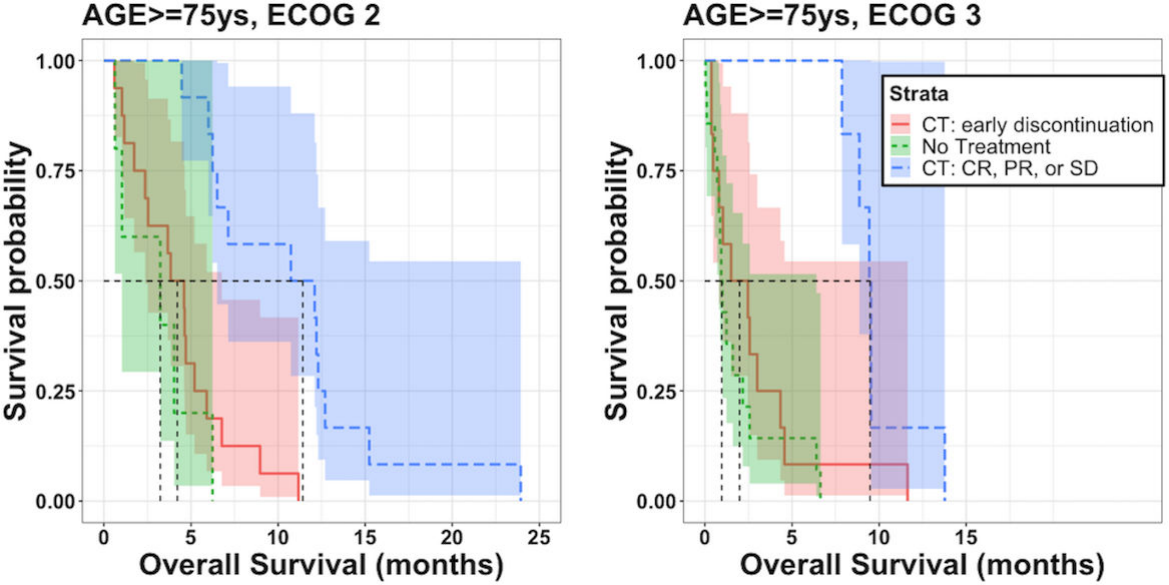
Kaplan-Meier curves of patients older than 75 years with ECOG performance status (PS) 2‐3 stratified by good response to first-line chemotherapy, early discontinuation of treatment, and patients who did not receive chemotherapy. CR: complete response; CT: chemotherapy; ECOG: Eastern Cooperative Oncology Group; PR: partial response; SD: stable disease.

When further stratifying the high-risk subgroups using the significant variables reported in Table 4, the subgroups showed similar survival to the nontreated subgroup. For example, Figure 4 shows the Kaplan-Meier curves of the comparative analysis for patients with ECOG PS 2‐3 and age ≥75 years.

## Discussion

In this study, we analyzed a longitudinal real-world cohort of patients with SCLC and their treatment pathways. The analysis allowed the study of the relationships between treatment decisions, phases of progression of disease, occurrence of AEs, and overall survival. To the best of our knowledge, this is one of the most detailed longitudinal datasets of SCLC real-world treatment decisions. This type of data aims to add novel insights on dosing effects over time, the emergence of toxicity, and the decision points relative to the clinical outcomes.

The improved prognosis of patients who experienced neutropenia aligns with previous findings [43,44]. The analysis allowed the investigation of treatment-related AEs in relation to the efficacy of therapy or to detect when this was instead linked to deterioration of the patient’s conditions. For example, the cumulative incidence curves of AEs stratified by responding patients against early discontinuation of the therapy in Figure 2 showed the role of neutropenia when the time windows of AEs may overlap with the efficacy of treatment (Figure 3).

We identified significant ORs relating to the dose of etoposide and increased occurrence of AEs. The analysis showed a similar OS for patients receiving 4 doses during first-line therapy, regardless of whether the patient had a dose adjustment/reduction or not (Figure 3). A worse prognosis was observed when there was a deterioration in ECOG PS or early discontinuation of treatment.

We interpret our findings as indicating that the occurrence of neutropenia reflects a longer duration of treatment exposure, rather than being a causal factor for improved survival. Patients who completed all 4 treatment cycles—often at reduced doses—demonstrated better outcomes than those receiving fewer cycles at higher doses. This is supported by the higher incidence of neutropenia among patients with treatment response, suggesting that extended exposure, not intensity, is the key contributor to therapeutic benefit.

SCLC treatment effects vary widely between individuals. This could suggest the need to identify key covariates and clinically relevant subgroups that might inform a more tailored dosing, or in some cases, guide decisions against treatment altogether, in order to improve survival while avoiding AEs in patients unlikely to benefit from therapy [11,12,45]. Indeed, etoposide dose calculation is based on BSA, which has been shown to present such limitations [46]. Similar analyses could be done for the second-line therapy. In fact, only a few patients received the full expected dose without dose reduction, and the platinum dose and PE treatment were strongly associated with AEs.

ECOG PS 2‐3 and elevated LDH serum levels are known to be prognostic factors of worse OS in SCLC [9,47]. Our results, obtained by the careful analysis of the longitudinal data line by line with the clinical experts, added a new insight by showing their role in predicting early discontinuation of treatment, which was associated with significantly poorer OS.

Another finding was the high probability of a favorable response to first-line therapy in male patients, along with their lower likelihood of experiencing AEs (Tables3  4; Tables S4 and S5 in Multimedia Appendix 1). Moreover, when stratifying older patients by sex, women who developed neutropenia exhibited survival rates comparable to those in the same subgroup who did not receive chemotherapy. A possible explanation is that female patients have historically been underrepresented in clinical trials [10,48,49]. In addition, in the second-line setting, the worse PFS and high frequency of early discontinuation for male patients might suggest the need for further studies regarding different therapy responses for males and females.

The high-risk subgroup analysis allowed detection of key groups of patients such as older patients with poor ECOG PS, where an eventual poor response to therapy is associated with similar survival as compared to patients not receiving chemotherapy. This finding is clinically relevant, as it highlights the importance of balancing the benefit and risk of treating such fragile patients and may help clinicians avoid overtreating this vulnerable population. These outcomes underline the potential role that real-world data may fill in providing evidence on treatment in groups of patients that are underrepresented in clinical trials [10].

The design of the analytic pipeline relied on the consideration that the data should be analyzed using models capable of capturing the complexity of the patient treatment pathways without sacrificing interpretability (such as black box machine learning models [25]). We believe that this is an important consideration when approaching real-world data. In fact, the discussions with the clinicians on the obtained results allowed the assessment of the validity of the results, the detection of potential inherent bias, and confounders related to operations and nonobservable effects not captured from the data. For example, it was observed that the 10-day dosing regimen of etoposide resulted in poorer survival. At the time of the present analysis, this dosing regimen had, however, already been abandoned due to toxicity caused by the limited time for patients to recover between the cycles (Tables2  3).

The study had several limitations. The reason for missing values was not at random for some of the variables, thus introducing some biases in the results. Particularly for baseline neutrophil counts, where missing observations were high across all treatment lines. This bias is partially mitigated by the MissForest algorithm because it is a nonparametric model, and the algorithm does not need an explicit assumption about the missing mechanism (ie, missing at random) [38].

Further, these variables were available only at the start of the treatment line, meaning that we were not able to analyze their time profiles (ie, neutrophil counts over time between doses). Moreover, it should be stated that AEs are not always described in medical notes, thus introducing investigator bias in the collected data.

Another limitation was the sample size of the high-risk subgroups and patients receiving the second-line treatment, resulting in higher estimates of variability of the outcomes as compared to the first-line treatment cohort. In detail, the odds ratios of the mixed effects models resulted in wide confidence intervals. The reason for this high variability in outcomes could be that these small cohorts were also associated with fewer longitudinal observations per patient (the number of administered doses). Increasing the sample sizes of these subgroups in the future by collecting more data will help to provide more precise estimates of the covariate effects. Moreover, patients receiving more than two lines were not included in the multivariate analysis, as this was only observed in a few instances.

The median age of our population was 72 years, yet we chose 75 years to better isolate a more vulnerable subset of patients by moving further from the median. This aligns with existing studies that identify this age group as particularly fragile, often requiring specialized considerations in treatment planning [50]. Future studies exploring different criteria and strategies to separate older patients would be required.

This was a single-center study, and expanding the analysis by including other SCLC cohorts from other centers would be beneficial. A comparison of the dose adaptation and the follow-up processes in clinical trials would be of high importance [10]. A future aspect to explore would be to expand the collection of longitudinal real-world data, designed similarly to the study presented here, in larger initiatives such as multicenter studies or cancer registries [51-53], and recent new proposed therapies (ie, durvalumab after chemoradiotherapy [54], tarlatamab/lubri in second- and third-line settings [55], and immune-oncology therapies [9]).

This study could inform the design of future works, such as multistate models of patient survival in function of different therapies and events [56,57], or model-informed precision dosing models to explore individualized treatment and dosing to reduce the risk of AEs (eg, neutropenia) without compromising the efficacy of the treatment in terms of survival outcomes [58,59]. Furthermore, the outcomes from the approaches presented herein can inform more reliable future analyses using, for example, machine learning algorithms suitable for modeling longitudinal processes, such as physics-based neural networks [60].

A continued fruitful collaboration between clinical and technical experts when analyzing complex data and contextualizing the clinical processes, as we did in this work, would enhance the impact of real-world data in clinical practice and clinical drug development.

## Supplementary material

10.2196/84042 Multimedia Appendix 1 Result tables.

10.2196/84042 Multimedia Appendix 2 Diagnostics and convergence assessment of Monte Carlo Markov Chains.

10.2196/84042 Multimedia Appendix 3 Sankey flows of the subsequent treatment lines and the dose adjustments.

## Abbreviations

AE: adverse event
BSA: body surface area
CNS: brain metastasis
CRP: C-reactive protein
csHR: cause-specific hazard ratio
CTCAE: Common Terminology Criteria for Adverse Events
ECOG PS: Eastern Cooperative Oncology Group performance status
GFR: glomerular filtration rate
HR: hazard ratio
IP: irinotecan platinum
LDH: lactate dehydrogenase
OR: odds ratio
OS: overall survival
PE: platinum etoposide
PFS: progression-free survival
RCT: randomized controlled trial
SCLC: small cell lung cancer
TNM: tumor node metastasis
