# Supplementary material for: Real-World Evidence of Treatment Outcomes in Small Cell Lung Cancer: A Bayesian Mixed Effects and Competitive Risk Approach
Source: JMIR Cancer. 2026 Apr 10;12:e84042. doi: 10.2196/84042 (PMC13070899; doi:10.2196/84042)
Supplement: Multimedia Appendix 2 [file cancer-v12-e84042-s002.docx]

Diagnostics and Convergence Assessment of Monte Carlo Markov Chains (MCMC)

In this section, we report trace plots, density plots, and summary tables with estimated, effective sample size, and Gelman-Rubin R-hat statistics of the Markov Chains Monte Carlo of the adverse effects models. The case reported are first-line therapy model with the full cohort, first-line therapy model for the fragile categories (as representative example we report the stage IVB subcohort), and second-line therapy full cohort.

For the first line therapy line with the full cohort, four chains of 2500 iterations per each are used. For the other sub-cohorts and the second line therapy 5000 iterations per each chain are used in order to ensure appropriate convergence.

# First-line therapy: whole cohort


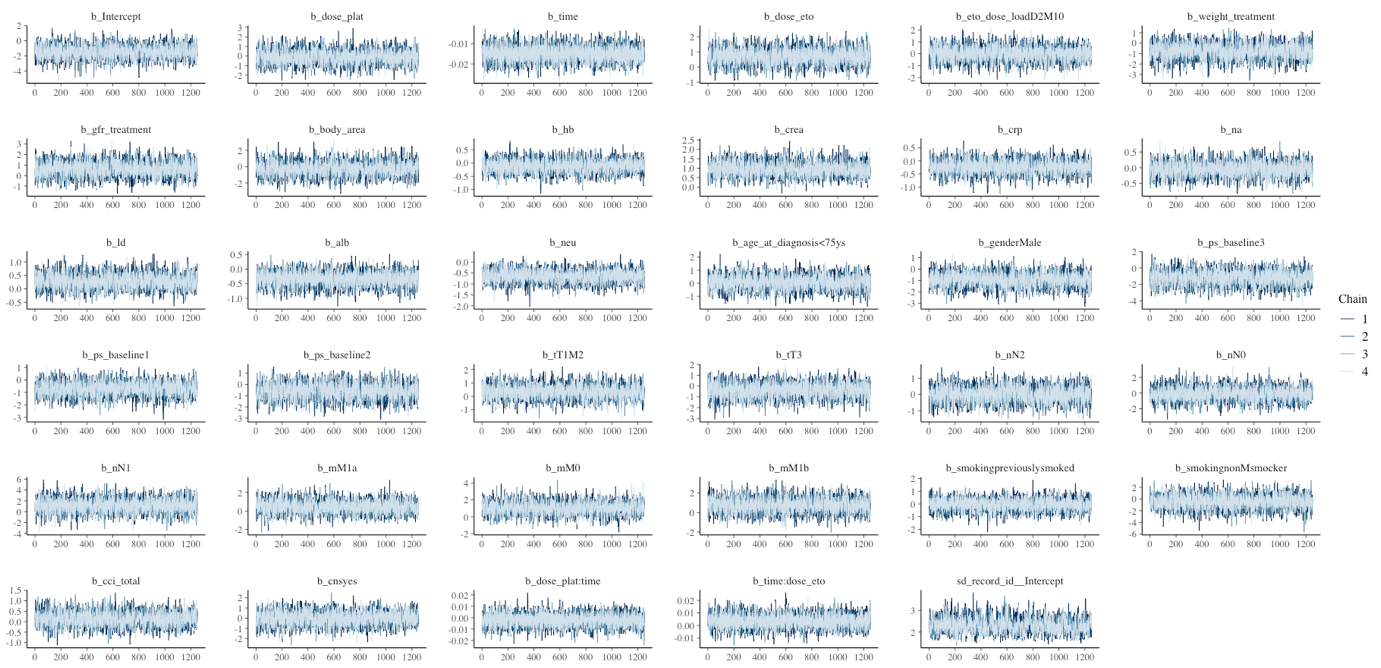


**Figure S1.** Trace plots.


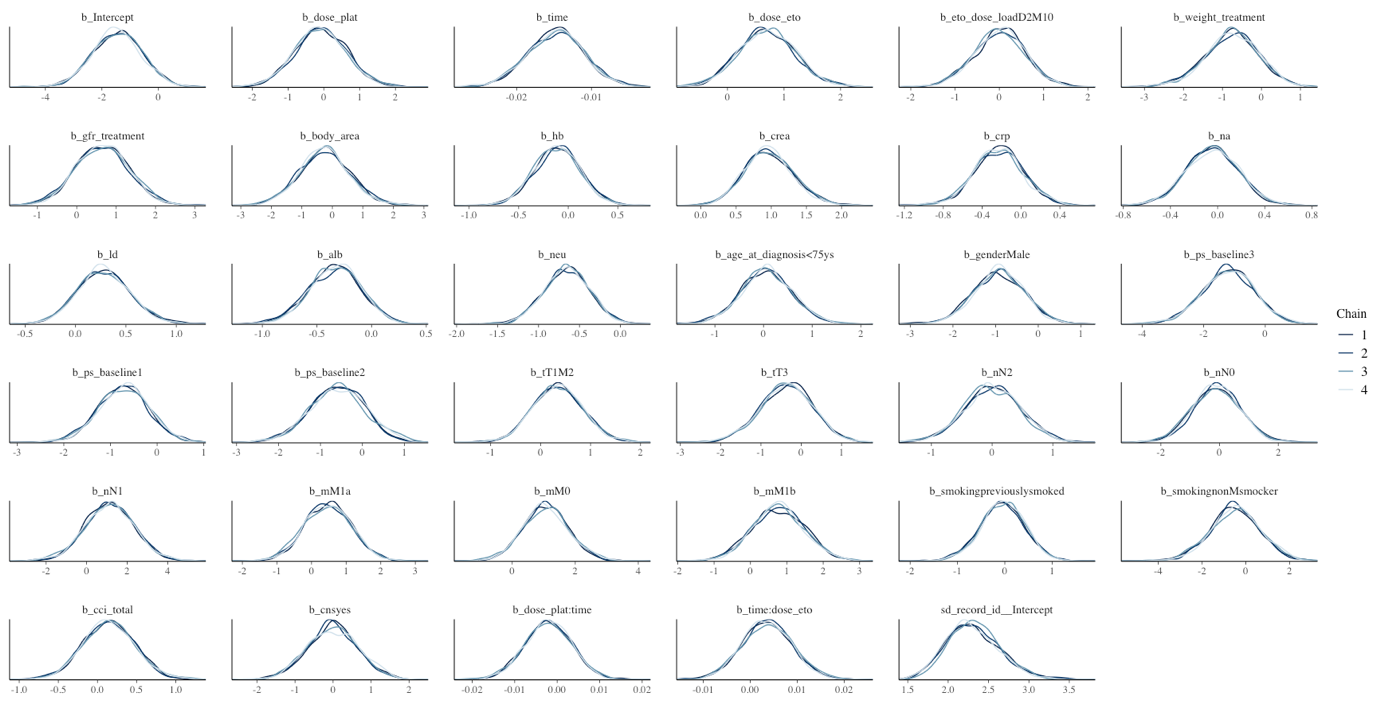


**Figure S2.** Density plots.

**Table S1.** Summary of estimates, Gelmann-Rubin statistics (R-hat) and bulk and tail effective sample size.

|  | **Estimate**  <dbl> | **Est.Error**  <dbl> | **l-95% CI**  <dbl> | **u-95% CI**  <dbl> | **Rhat**  <dbl> | **Bulk_ESS**  <dbl> | **Tail_ESS**  <dbl> |
| --- | --- | --- | --- | --- | --- | --- | --- |
| Intercept | -1.453343910 | 0.873971167 | -3.149468060 | 0.229392998 | 1.0007257 | 3389.578 | 3676.872 |
| dose_plat | -0.092923135 | 0.724985916 | -1.505505559 | 1.353564703 | 1.0014874 | 2926.067 | 3236.107 |
| time | -0.014768962 | 0.003796998 | -0.022334896 | -0.007292122 | 1.0001644 | 8186.747 | 4686.485 |
| dose_eto | 0.696422926 | 0.506071516 | -0.275606083 | 1.726199732 | 1.0007414 | 3154.308 | 3509.413 |
| eto_dose_loadD2M10 | 0.002218417 | 0.587267947 | -1.167433359 | 1.147889776 | 1.0008300 | 3448.329 | 3528.234 |
| weight_treatment | -0.766786915 | 0.702646698 | -2.278941419 | 0.553516151 | 0.9998915 | 3495.122 | 3361.284 |
| gfr_treatment | 0.647688918 | 0.683680735 | -0.684977829 | 1.988883440 | 1.0011629 | 2766.858 | 3363.559 |
| body_area | -0.248220225 | 0.842780327 | -1.896496260 | 1.455335425 | 1.0005121 | 3196.401 | 3560.827 |
| hb | -0.111273526 | 0.235811825 | -0.572044244 | 0.362766747 | 1.0002903 | 3609.524 | 3943.944 |
| crea | 0.966629857 | 0.354249678 | 0.311213964 | 1.700064149 | 1.0012910 | 3614.892 | 3260.811 |
| crp | -0.230571979 | 0.257843418 | -0.753793908 | 0.277683917 | 1.0008479 | 3507.023 | 3054.552 |
| na | -0.045057171 | 0.225543179 | -0.503343520 | 0.389764329 | 1.0004167 | 3828.929 | 3437.858 |
| ld | 0.280599615 | 0.263947895 | -0.218028509 | 0.814015679 | 1.0004581 | 3609.008 | 3866.854 |
| alb | -0.334870487 | 0.239368824 | -0.814625673 | 0.120582860 | 1.0006827 | 4672.371 | 3547.130 |
| neu | -0.656396535 | 0.280978814 | -1.206720548 | -0.113277276 | 1.0005653 | 3403.185 | 3748.437 |
| age_at_diagnosis<75ys | 0.061815389 | 0.529720923 | -0.982795225 | 1.105378332 | 1.0004374 | 3740.348 | 3128.165 |
| genderMale | -0.919923390 | 0.596387193 | -2.094566424 | 0.252521443 | 1.0007870 | 3417.767 | 3419.577 |
| ps_baseline3 | -1.198496273 | 0.852058709 | -2.910146794 | 0.441160034 | 0.9997613 | 3662.471 | 3809.203 |
| ps_baseline1 | -0.723822098 | 0.548007579 | -1.811460307 | 0.334151131 | 1.0016005 | 3111.525 | 3703.829 |
| ps_baseline2 | -0.589796937 | 0.668290048 | -1.931071381 | 0.721057783 | 1.0013315 | 2966.955 | 3386.243 |
| tT1M2 | 0.342560976 | 0.509244853 | -0.648333612 | 1.343515685 | 1.0007942 | 3721.806 | 3378.413 |
| tT3 | -0.369693965 | 0.660314360 | -1.703399616 | 0.898376659 | 1.0000229 | 4233.087 | 3942.952 |
| nN2 | 0.002613499 | 0.470039775 | -0.943490065 | 0.904225136 | 1.0008813 | 3021.503 | 4037.567 |
| nN0 | -0.097345510 | 0.830468764 | -1.684911192 | 1.517661571 | 1.0003786 | 3958.572 | 3302.109 |
| nN1 | 1.137579025 | 1.231797140 | -1.320830851 | 3.539234850 | 1.0002001 | 4235.970 | 3884.360 |
| mM1a | 0.476650169 | 0.680122013 | -0.853587027 | 1.817884904 | 1.0001435 | 3671.893 | 3730.122 |
| mM0 | 1.074551837 | 0.736650879 | -0.357109220 | 2.569887052 | 1.0007814 | 2983.799 | 3408.597 |
| mM1b | 0.765576401 | 0.726459100 | -0.631197561 | 2.192439557 | 1.0015214 | 3698.934 | 3695.033 |
| smokingpreviouslysmoked | -0.046889059 | 0.467694713 | -1.000868122 | 0.853165234 | 1.0005882 | 3913.169 | 3868.575 |
| smokingnonMsmocker | -0.480305456 | 1.152345579 | -2.756181075 | 1.767885954 | 1.0008530 | 4158.895 | 3742.752 |
| cci_total | 0.147349492 | 0.334632051 | -0.500213837 | 0.801644205 | 1.0008069 | 3213.733 | 3297.387 |
| cnsyes | -0.010037499 | 0.668916436 | -1.323662853 | 1.308187521 | 1.0032546 | 3507.340 | 3564.256 |
| dose_plat:time | -0.002108888 | 0.005540663 | -0.013106599 | 0.008670917 | 1.0006773 | 7739.626 | 4262.017 |
| time:dose_eto | 0.003592342 | 0.005365524 | -0.006788768 | 0.014183678 | 1.0006648 | 6829.787 | 3992.132 |

# First-line therapy: fragile subgroups

## Stage IVB


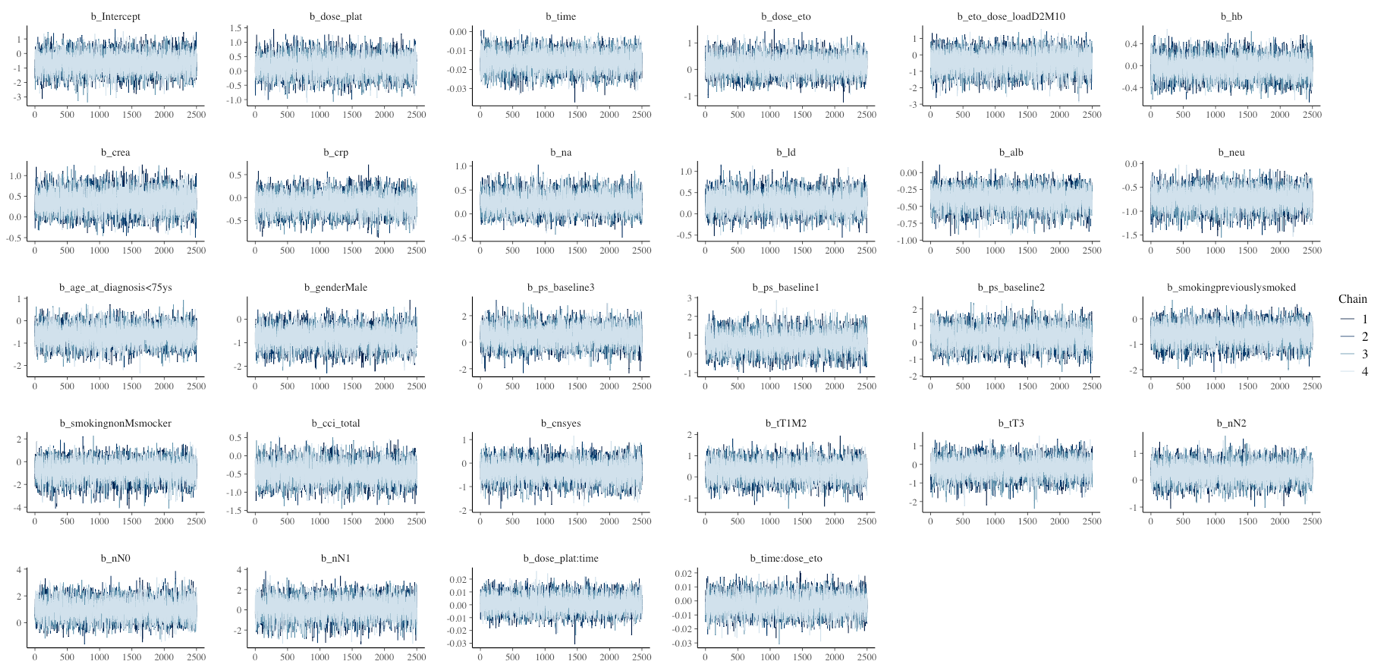


**Figure S3.** Trace plots.


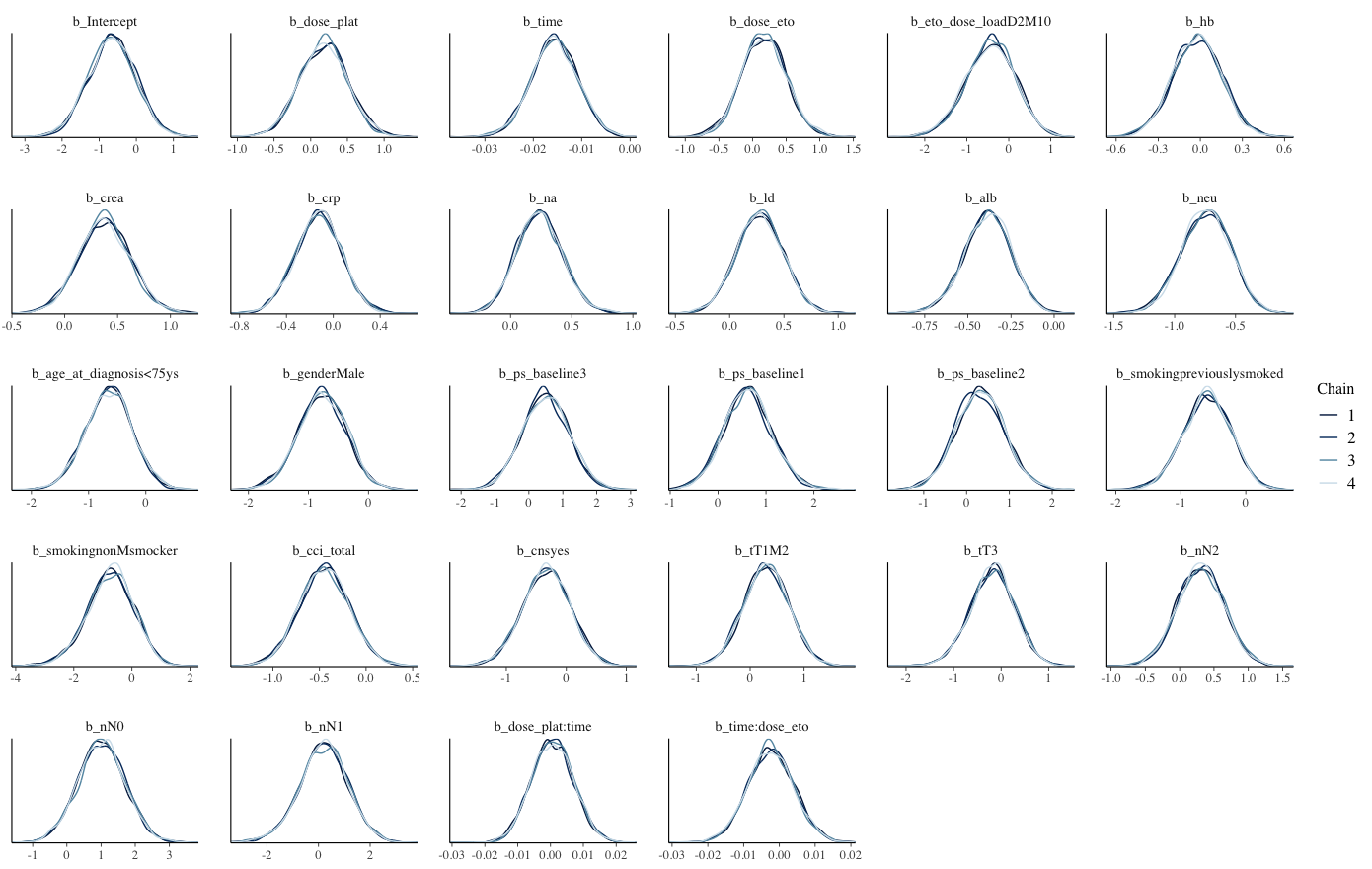


**Figure S4.** Density plots.

**Table S2.** Summary of estimates, Gelmann-Rubin statistics (R-hat) and bulk and tail effective sample size.

|  | **Estimate**  <dbl> | **Est.Error**  <dbl> | **l-95% CI**  <dbl> | **u-95% CI**  <dbl> | **Rhat**  <dbl> | **Bulk_ESS**  <dbl> | **Tail_ESS**  <dbl> |
| --- | --- | --- | --- | --- | --- | --- | --- |
| Intercept | -0.6572954006 | 0.644715048 | -1.93202097 | 0.594637394 | 1.0006876 | 7786.365 | 7502.275 |
| dose_plat | 0.1751290363 | 0.328538360 | -0.46468662 | 0.814535753 | 1.0009371 | 7230.898 | 7870.062 |
| time | -0.0156383201 | 0.004801020 | -0.02523535 | -0.006436943 | 1.0002362 | 14565.550 | 7983.046 |
| dose_eto | 0.1670997507 | 0.333762409 | -0.49238978 | 0.828170867 | 1.0006104 | 7268.540 | 6919.874 |
| eto_dose_loadD2M10 | -0.4385032470 | 0.591602617 | -1.65077324 | 0.668735040 | 1.0004974 | 11066.648 | 8281.247 |
| hb | -0.0213107126 | 0.176281913 | -0.37265980 | 0.324757202 | 1.0004277 | 11999.438 | 7896.134 |
| crea | 0.3769803314 | 0.245276957 | -0.10214742 | 0.867860794 | 1.0009009 | 9410.153 | 7753.475 |
| crp | -0.1260095720 | 0.198469343 | -0.51640574 | 0.258303999 | 1.0004516 | 8689.848 | 8256.238 |
| na | 0.2378980829 | 0.188754172 | -0.12277330 | 0.618330325 | 0.9999230 | 9488.122 | 7807.135 |
| ld | 0.2784286236 | 0.217387901 | -0.14063636 | 0.701496265 | 1.0000988 | 8030.382 | 8236.287 |
| alb | -0.3881717408 | 0.138451961 | -0.67717487 | -0.126148604 | 1.0002653 | 9950.499 | 7775.124 |
| neu | -0.7439935538 | 0.204375534 | -1.15297474 | -0.356646512 | 1.0001899 | 8803.041 | 7895.335 |
| age_at_diagnosis<75ys | -0.6458985726 | 0.409204113 | -1.45416077 | 0.149806355 | 1.0004249 | 11424.357 | 8024.635 |
| genderMale | -0.7712234808 | 0.400601386 | -1.59352917 | -0.002886001 | 1.0006969 | 10482.063 | 7420.182 |
| ps_baseline3 | 0.4906610261 | 0.689038383 | -0.85551217 | 1.832504190 | 1.0007251 | 6708.661 | 7561.933 |
| ps_baseline1 | 0.6185458776 | 0.508381032 | -0.36992103 | 1.638812862 | 1.0004399 | 5464.357 | 7545.320 |
| ps_baseline2 | 0.3134879561 | 0.572469586 | -0.80719828 | 1.459220281 | 1.0004956 | 5686.767 | 7159.720 |
| smokingpreviouslysmoked | -0.5988306239 | 0.367659760 | -1.33607926 | 0.094910042 | 1.0002206 | 11196.092 | 7450.574 |
| smokingnonMsmocker | -0.7526493121 | 0.815071244 | -2.45307784 | 0.754528200 | 1.0002643 | 12314.486 | 7637.531 |
| cci_total | -0.4478408424 | 0.270363646 | -0.97751700 | 0.083793622 | 0.9998983 | 10861.186 | 8137.643 |
| cnsyes | -0.3345315272 | 0.399605513 | -1.13360078 | 0.438224622 | 1.0007777 | 9522.194 | 7203.351 |
| tT1M2 | 0.2970542593 | 0.423202369 | -0.54289076 | 1.112939514 | 1.0000176 | 9162.818 | 8407.395 |
| tT3 | -0.1895587045 | 0.476372885 | -1.15738298 | 0.717980451 | 1.0004054 | 13841.936 | 8105.431 |
| nN2 | 0.3073644538 | 0.341111075 | -0.36644987 | 0.970201096 | 1.0001653 | 12065.459 | 7974.078 |
| nN0 | 1.0146636636 | 0.687950783 | -0.34611587 | 2.353607045 | 0.9998684 | 8595.471 | 8029.994 |
| nN1 | 0.1937711314 | 0.945925994 | -1.77522081 | 2.013431476 | 1.0003076 | 11565.355 | 7146.626 |
| dose_plat:time | 0.0006401852 | 0.006375390 | -0.01152045 | 0.013329841 | 1.0003006 | 8430.519 | 8128.468 |
| time:dose_eto | -0.0023848430 | 0.006497748 | -0.01520430 | 0.010492629 | 1.0003197 | 7581.221 | 7596.465 |

# Second-line therapy: whole cohort


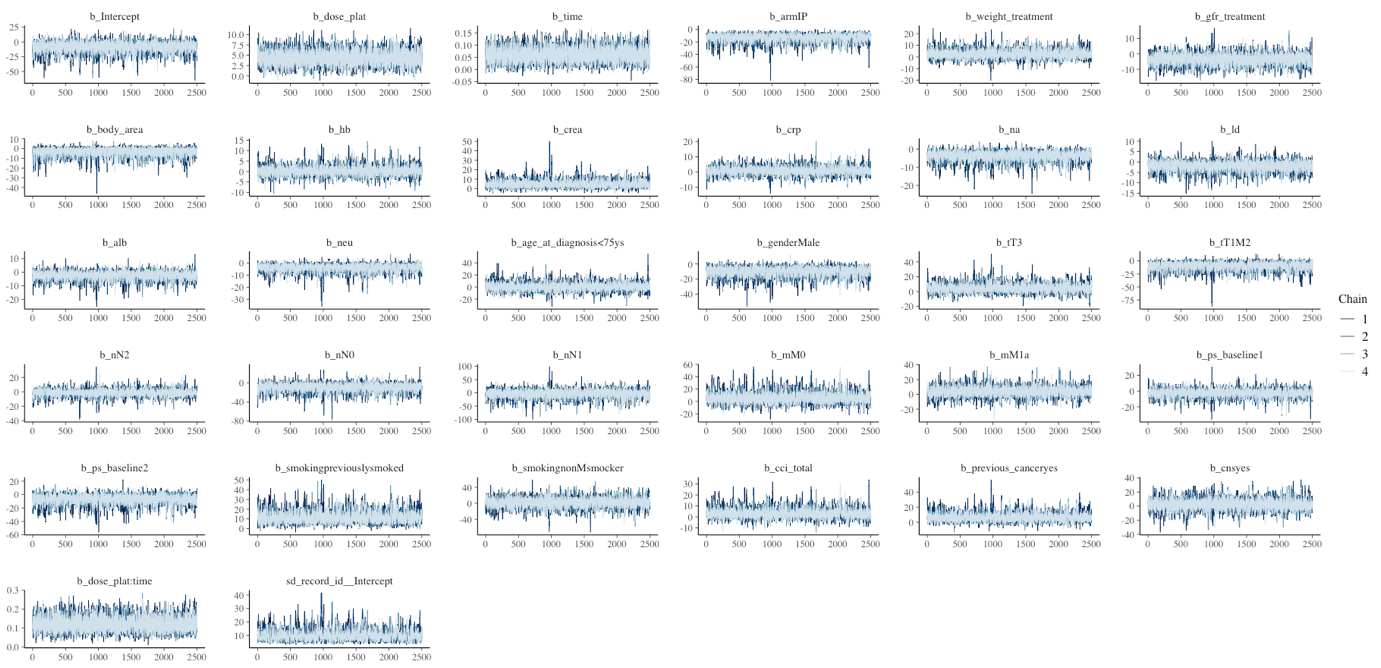


**Figure S5.** Trace plots.


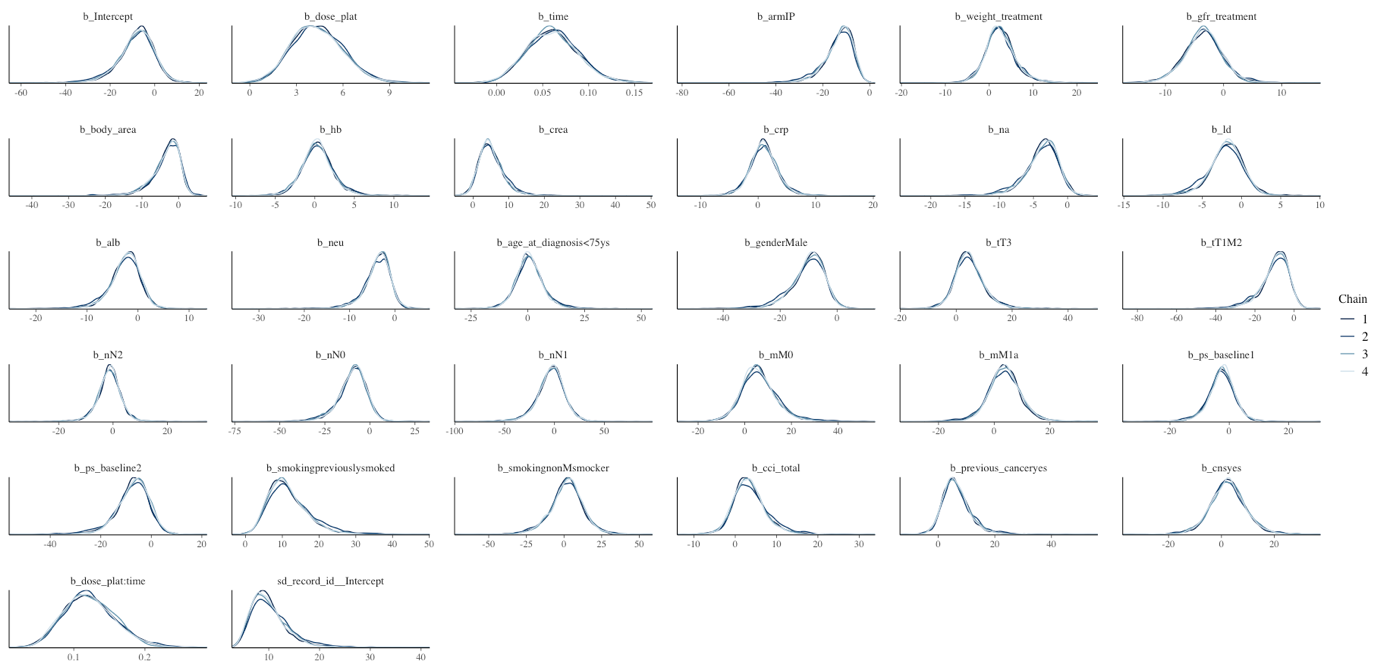


**Figure S6.** Density plots.

**Table S3.** Summary of estimates, Gelmann-Rubin statistics (R-hat) and bulk and tail effective sample size.

|  | **Estimate**  <dbl> | **Est.Error**  <dbl> | **l-95% CI**  <dbl> | **u-95% CI**  <dbl> | **Rhat**  <dbl> | **Bulk_ESS**  <dbl> | **Tail_ESS**  <dbl> |
| --- | --- | --- | --- | --- | --- | --- | --- |
| Intercept | -8.30022193 | 8.35754518 | -27.430983924 | 5.8592220 | 1.001575 | 3588.634 | 2872.869 |
| dose_plat | 4.34884002 | 1.65307759 | 1.334993537 | 7.7768254 | 1.000851 | 4612.675 | 6465.659 |
| time | 0.06095496 | 0.02804935 | 0.009746333 | 0.1198998 | 1.000216 | 6939.167 | 7005.325 |
| armIP | -13.47833571 | 6.39117797 | -28.809130833 | -4.1842770 | 1.003399 | 1680.689 | 1809.314 |
| weight_treatment | 2.59929735 | 3.58689775 | -4.017671282 | 10.5922132 | 1.000806 | 3636.498 | 2579.287 |
| gfr_treatment | -3.50468383 | 3.38947874 | -10.343771792 | 3.1829514 | 1.000785 | 3750.347 | 3406.539 |
| body_area | -3.39952678 | 4.00273873 | -13.055337141 | 2.1827302 | 1.001352 | 4326.320 | 2269.427 |
| hb | 0.37812543 | 2.14423236 | -3.612577398 | 4.9764088 | 1.001153 | 3767.632 | 2556.229 |
| crea | 5.16224029 | 3.79164462 | -0.599565892 | 13.7543840 | 1.001085 | 2106.270 | 2198.898 |
| crp | 0.96110270 | 2.55583236 | -4.103538402 | 6.2866937 | 1.001842 | 3494.562 | 3387.040 |
| na | -3.82775604 | 2.55750120 | -9.939785857 | 0.2272613 | 1.002056 | 1879.890 | 2010.921 |
| ld | -2.00542804 | 2.29331385 | -7.124712354 | 2.0754991 | 1.003117 | 3300.841 | 3107.985 |
| alb | -2.52244802 | 3.04799229 | -9.363609211 | 2.6603343 | 1.001628 | 2826.769 | 2125.514 |
| neu | -3.94509552 | 3.07320933 | -11.095934502 | 0.8197625 | 1.001644 | 2500.785 | 2235.506 |
| age_at_diagnosis<75ys | 0.78384497 | 6.32921908 | -11.194591417 | 14.4963931 | 1.001517 | 3349.587 | 3020.544 |
| genderMale | -10.36855194 | 6.15499909 | -24.701876394 | -0.6991992 | 1.001919 | 2055.951 | 2134.479 |
| tT3 | 4.78820194 | 5.44847909 | -4.871168962 | 16.9176548 | 1.000880 | 3695.945 | 2964.947 |
| tT1M2 | -10.03124646 | 7.57562750 | -28.354614788 | 1.3099453 | 1.002461 | 2480.743 | 1946.767 |
| nN2 | -1.38323895 | 4.55622366 | -10.882925336 | 7.2692915 | 1.000915 | 3717.551 | 2986.035 |
| nN0 | -9.75102221 | 8.27189477 | -28.732383935 | 4.2905810 | 1.000852 | 3817.682 | 3593.837 |
| nN1 | -3.70092635 | 14.50054500 | -35.180887926 | 22.9875020 | 1.001380 | 4882.164 | 3556.140 |
| mM0 | 6.22438310 | 8.11912407 | -8.007884945 | 24.5382253 | 1.001854 | 3235.308 | 3216.426 |
| mM1a | 3.76754631 | 6.26827041 | -8.427952654 | 16.5056574 | 1.001564 | 4097.171 | 3519.057 |
| ps_baseline1 | -2.68568410 | 4.75701909 | -13.041693511 | 6.1638888 | 1.001432 | 3401.757 | 3382.014 |
| ps_baseline2 | -7.48624824 | 6.88396399 | -23.922910617 | 3.8646157 | 1.002958 | 2406.779 | 2048.486 |
| smokingpreviouslysmoked | 11.75635765 | 5.72528193 | 3.290363428 | 25.4141186 | 1.003090 | 1889.028 | 2128.046 |
| smokingnonMsmocker | 1.78211272 | 11.47684830 | -22.969759105 | 24.1086336 | 1.001409 | 4323.902 | 3618.930 |
| cci_total | 3.59244201 | 4.25885323 | -3.544385695 | 13.3857608 | 1.003201 | 2726.774 | 2546.063 |
| previous_canceryes | 6.59574072 | 5.36601712 | -1.880081537 | 19.4098944 | 1.000924 | 2439.827 | 2003.677 |
| cnsyes | 2.47009816 | 7.19617516 | -11.929965375 | 17.3171145 | 1.000224 | 4396.225 | 4503.747 |
| dose_plat:time | 0.12259563 | 0.03648388 | 0.057931996 | 0.1994876 | 1.000060 | 5353.479 | 5955.556 |
