# Supplementary material for: Real-World Evidence of Treatment Outcomes in Small Cell Lung Cancer: A Bayesian Mixed Effects and Competitive Risk Approach
Source: JMIR Cancer. 2026 Apr 10;12:e84042. doi: 10.2196/84042 (PMC13070899; doi:10.2196/84042)
Supplement: Multimedia Appendix 3 [file cancer-v12-e84042-s003.docx]

Sankey Flows of the Subsequent Treatment Lines and the Dose Adjustments


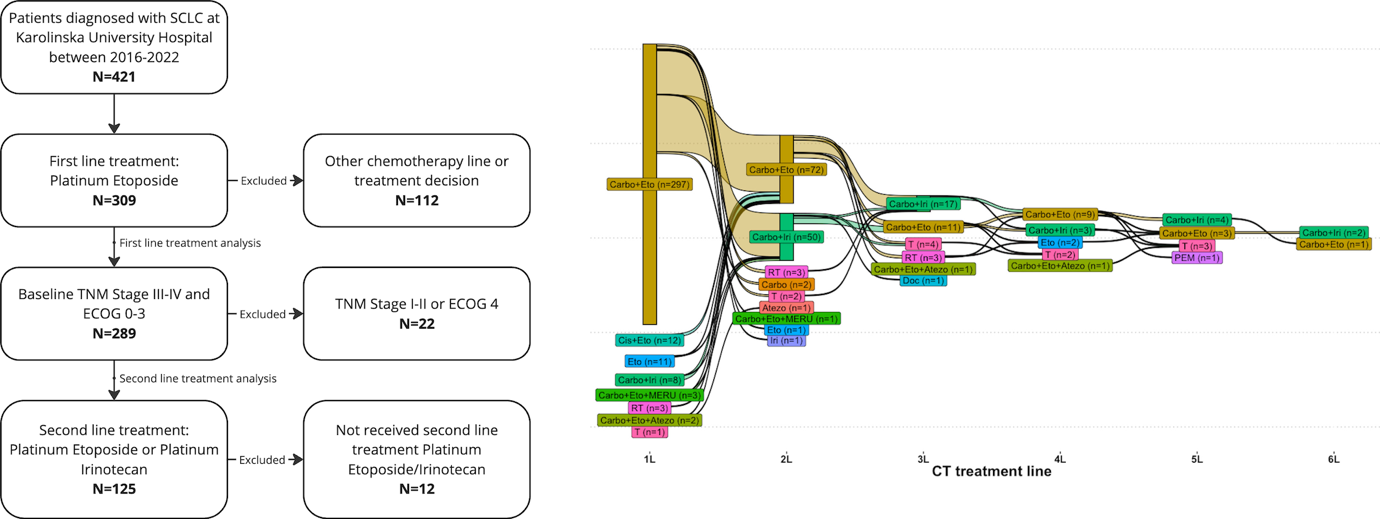


**Figure S1.** Sankey flow of the subsequent chemotherapy treatment lines.


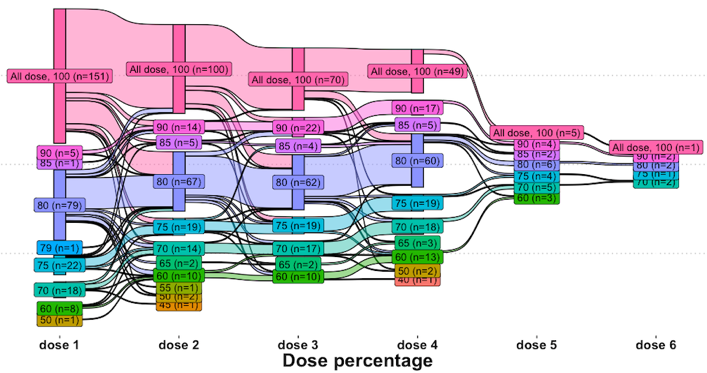


**Figure S2.** Sankey flow or the subsequent doses percentages for patients receiving first-line platinum etoposide.


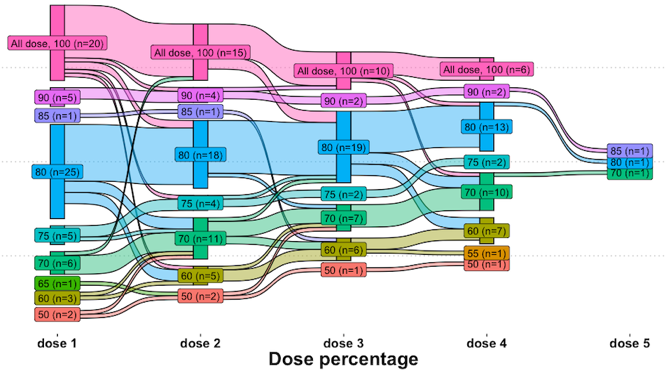


**Figure S3.** Sankey flow or the subsequent doses percentages for patients receiving second-line platinum etoposide after first-line platinum etoposide.


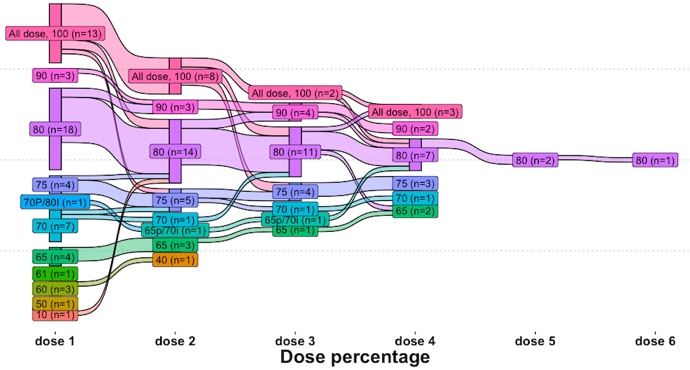


**Figure S4.** Sankey flow or the subsequent doses percentages for patients receiving second-line platinum irinotecan after first-line platinum etoposide.
